# A Review on the Adaption of Alginate-Gelatin Hydrogels for 3D Cultures and Bioprinting

**DOI:** 10.3390/ma14040858

**Published:** 2021-02-10

**Authors:** Magdalena B. Łabowska, Karolina Cierluk, Agnieszka M. Jankowska, Julita Kulbacka, Jerzy Detyna, Izabela Michalak

**Affiliations:** 1 Department of Mechanics, Materials and Biomedical Engineering, Faculty of Mechanical Engineering, Wroclaw University of Science and Technology, Smoluchowskiego 25, 50-370 Wroclaw, Poland; magdalena.labowska@pwr.edu.pl (M.B.Ł); agnieszka.jankowska@pwr.edu.pl (A.M.J.); 2 Faculty of Chemistry, Wroclaw University of Science and Technology, Norwida 4/6, 50-373 Wroclaw, Poland; karolinacierluk@gmail.com; 3 Department of Molecular and Cellular Biology, Faculty of Pharmacy, Wroclaw Medical University, Borowska 211A, 50-556 Wroclaw, Poland; julita.kulbacka@umed.wroc.pl; 4 Department of Advanced Material Technologies, Faculty of Chemistry, Wroclaw University of Science and Technology, Smoluchowskiego 25, 50-370 Wroclaw, Poland; izabela.michalak@pwr.edu.pl

**Keywords:** alginate, gelatin, hydrogels, cell cultures, bioprinting

## Abstract

Sustaining the vital functions of cells outside the organism requires strictly defined parameters. In order to ensure their optimal growth and development, it is necessary to provide a range of nutrients and regulators. Hydrogels are excellent materials for 3D in vitro cell cultures. Their ability to retain large amounts of liquid, as well as their biocompatibility, soft structures, and mechanical properties similar to these of living tissues, provide appropriate microenvironments that mimic extracellular matrix functions. The wide range of natural and synthetic polymeric materials, as well as the simplicity of their physico-chemical modification, allow the mechanical properties to be adjusted for different requirements. Sodium alginate-based hydrogel is a frequently used material for cell culture. The lack of cell-interactive properties makes this polysaccharide the most often applied in combination with other materials, including gelatin. The combination of both materials increases their biological activity and improves their material properties, making this combination a frequently used material in 3D printing technology. The use of hydrogels as inks in 3D printing allows the accurate manufacturing of scaffolds with complex shapes and geometries. The aim of this paper is to provide an overview of the materials used for 3D cell cultures, which are mainly alginate–gelatin hydrogels, including their properties and potential applications.

## 1. Introduction

Hydrogels are three-dimensionally cross-linked networks of hydrophilic polymeric materials capable of absorbing large amounts of liquid between their polymeric chains. These materials are created through the reaction of one or more monomers or by association bonds (hydrogen bonds and strong van der Waals forces between chains). The ability of these materials to attract water molecules and their tendency to dissolve in water result from the presence of hydrophilic functional groups connected with the polymeric backbone (−OH, −COOH, −COO, > C = O, −CHNH_2_, −CONH, −CONH_2_, −NH_2_, and −SO_3_H) [1,2,3,4,5,6]. The amount of water that polymer networks of hydrogels are capable of absorbing is between 10% and 1000 times greater than dry weight [7]. However, the content—and thus the volume—of a hydrogel can change over time, depending on the external parameters (e.g., the nature of the solvent, temperature, pH), as well as on the drying process [5].

**Figure 1:**
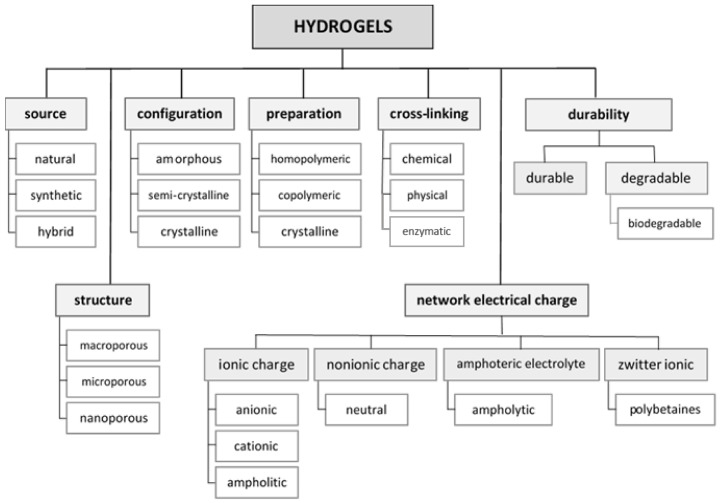
Classification of hydrogels.

There are numerous literature reports on the classification of hydrogels. They can be grouped according to their source, configuration (amorphous, semi-crystalline, crystalline) [2,3,8,9], type of cross-linking (chemically cross-linked, physically cross-linked, enzymatically cross-linked) [2,10], and polymeric composition (homopolymeric, copolymeric, multipolymer interpenetrating polymeric—which consists of at least two independent cross-linked polymers (synthetic or natural), however they are not covalently bonded to each other) [2,9,11]. Hydrogels can also be divided according to their durability. Despite their high endurance and stability in swelling environments, durable and degradable hydrogels can be distinguished. In comparison to natural sources of hydrogels, synthetic sources have better mechanical strength but a slower degradation rate. However, they are not biocompatible in vivo, unlike natural hydrogels [9,12,13]. Hydrogels can also be divided depending on the presence or absence of electrical charge in the polymeric chain. Hydrogels that have no charge in their backbone are non-ionic or neutral. Conversely, ionic hydrogels are cationic, which are positive-charge-bearing, or anionic, which have a negative charge in their chains. The presence of a charge in the backbones affects their dissociation in different media environments, i.e., cationic hydrogels swell in media at lower pH (< 7) and anionic hydrogels at higher pH (> 7) [14]. There is also a group of ampholyte hydrogels that contain both cationic and anionic functional groups. These are characterized by their hydrophilicity and good biocompatibility [8,9,15]. The classification of hydrogels is summarized in Figure 1.

The materials used in the hydrogel manufacturing process include both natural and synthetic polymers, as well as hybrid hydrogels, which involve combinations of natural and synthetic polymers [2,12]. These natural polymers can be obtained from natural sources such as plants, bacterial cultures, or animals, including polysaccharides (e.g., agarose, alginate, carrageenan, chitosan), glycosaminoglycans (e.g., heparin, hyaluronic acid, keratan sulfate), and polypeptides or proteins (e.g., collagen, fibrin, gelatin, silk). Examples of synthetic polymers used to manufacture hydrogels include poly(ethylene glycol) (PEG), poly(2-hydroxyethyl methacrylate) (PHEMA), and poly(vinyl alcohol) (PVA) [1,16,17]. Hydrogels can occur in different forms, such as solid, semi-solid, and liquid states. These states depend on various factors, such as the chemical compositions of the materials, the amounts and types of solvents, the nature of the bonds, the polarity of the polymers, the charges, and the temperature. However, through physico-chemical modification of the structure and cross-linking, it is possible to obtain hydrogels with desired viscous, rheological, and mechanical properties [4,5,18].

The universality of hydrogels enables their application as biosensors in the food industry, agriculture, water purification, diagnostics, cosmetics, dentistry, and biomedical fields (e.g., in pharmaceuticals, drug delivery, and wound dressings) [2,10,19]. In bioengineering, they are mainly utilized due to their biocompatibility, biodegradability, and low immunogenicity. The water content in hydrogels supports their integrity, solubility, and diffusion of their substances, which are useful factors in delivering a therapeutic agent. The properties of hydrogels, such as their soft structures, elasticity, and similarity to living tissues, make them excellent for tissue engineering and regenerative medicine applications, as well as in stem cell cultures and cell implantation. Tissues and organs in the human body are characterized by their unique structures, differing in water content, as well as their mineral and protein contents, therefore it is necessary to use a biopolymer to manufacture a hydrogel with common building blocks [2,5,6,12]. Hydrogels are excellent materials for the production of substrates for cell cultures, for example three-dimensional porous scaffolds that can imitate the extracellular matrix (ECM), ensuring cell proliferation and differentiation in the in vitro microenvironment. Furthermore, their mechanical and structural properties allow imitation of native tissues and provide cell protection against possible damage caused by external factors [6,17,20].

In contrast to 2D cell cultures, 3D structures provide more space for cell proliferation and more closely mimic the natural environment [17]. The use of hydrogel materials for 3D cell cultures involves significant challenges, such as the in vitro behavior of the cells and the influence of external conditions on their appropriate development. Although each cell line has different requirements and the preparation of a universal in vivo environment is not possible, the simplicity with which hydrogel properties can be modified enables them to be customized according to the specific requirements. Furthermore, the utilization in the biomedical field of 3D printing technology makes it possible to achieve more accurate geometries and shapes for the manufactured structures [21,22]. Three-dimensional printing is a versatile technique for creating complex biomimetic tissue structures with extraordinary spatial precision [23].

The aim of this review is to present trends in the utilization of alginate–gelatin hydrogels as substrates in 3D cell cultures, as well as an overview of their material characteristics. Due to the lack of biological activity, sodium alginate cannot be employed alone for the production of hydrogel matrices. It is most often used in combination with other polymers to allow the adhesion of cells and their healthy development. The combination of sodium alginate and gelatin provides an excellent hydrogel for use as a substrate for 3D cell cultures due to its unique biological properties, such as its biocompatibility, biodegradability, and non-toxicity. Some of the recent additions to this field are cellulose nanomaterials, which improve the chemical, physical, and biological properties of the created hydrogels. The easily modifiable mechanical properties of these materials can be adapted to the living tissue, making them ideal environments for cell culture development. Moreover, the rheological properties of alginate-gelatin hydrogels make them excellent candidates for utilization in additive technologies at room temperature. Thus, it is possible to use them as hydrogel inks within the cell content, without risking damage.

## 2. Materials and Methods for the Preparation and Characterization of Hydrogel Substrates

The most important characteristics of a hydrogel are its permeability and water retention capacity. In contact with water, the polar hydrophilic groups are hydrated, which leads to the creation of primary bound water and swelling of the network. This results in the exposure of hydrophobic groups capable of interacting with water molecules and forming secondary bound water. Often total bound water is also formed by combining primary and secondary bound water. The hydrogel reaches a balanced swelling level by creating an elastic network retracting force by opposing covalent or physical cross-linking [24,25].

The biocompatibility of a hydrogel depends on the interaction of the material and its degradation products with living tissue or body fluids—it should not induce toxic or immunological responses. The low interfacial free energy of the hydrophilic surface in contact with body fluids results in a low tendency of proteins and cells to adhere to these surfaces, which makes the hydrogel satisfactorily biocompatible [26,27]. Hydrogels are characterized by their flexibility and soft structures. Therefore, these hydrated polymeric biomaterials are often utilized in biomedical fields as ECM substitutes. The biological, physical, and mechanical properties, as well as the permeability control of hydrogels, provide a biomimetic microenvironment similar to that of living tissue. The physico-chemical viscosity modification of a hydrogel allows control of its mechanical properties, as well as enabling various forms of the material to be achieved for 3D cell cultures, such as beads, injectable gels, moldable gels, and macroporous structures [28,29]. A hydrogel’s porosity is a very important aspect, because large surface areas foster attachment and ingrowth of cells. Moreover, pores connected by channels enable transport of gases, nutrients, and waste [20].

Hydrogel cell culture substrates can be prepared as mono- or multi-component mixtures of different polymers ratios. Composite substrates are preferred due to their ability to combine the functions of the individual components, as opposed to single-component substrates [30]. Among the natural polymers used for hydrogel fabrication for 3D cell cultures are alginate, collagen, gelatin, and fibrin. One of the most popular materials is based on sodium alginate [29,31]. Alginate hydrogel is an excellent material for imitating the extracellular matrix, which is the outer environment of the cell. However, this natural polymer does not provide mammalian cell-adhesive ligands, i.e., short polypeptide sequences in ECM proteins, which mimic ligands used in integrin-mediating cell adhesion formation and facilitate cell attachment [32,33]. In order to form three-dimensional structures in hydrogels for cells, components that are part of the in vivo matrix are required. On the surfaces of cells are receptors, which allow the cells to bind with matrix ligands. Typically, cells have receptors for many different ECM factors, which allow them to simultaneously bind to various ligands and to form networks. The cells themselves also produce components of the extracellular matrix; nevertheless, supplementation of hydrogels with ligands is required [34]. Thus, to mimic the ECM, this material is utilized in combination with other materials, such as gelatin, chitosan [32], elastin, agarose, or hyaluronic acid [20]. Moreover, additives such as nanocellulose act as reinforcing agents in hydrogel materials, increasing the mechanical properties and improving the printability [35]. In this review, we would like to focus mainly on alginate–gelatin hydrogels for cell cultures.

### 2.1. Alginate

**Figure 2:**
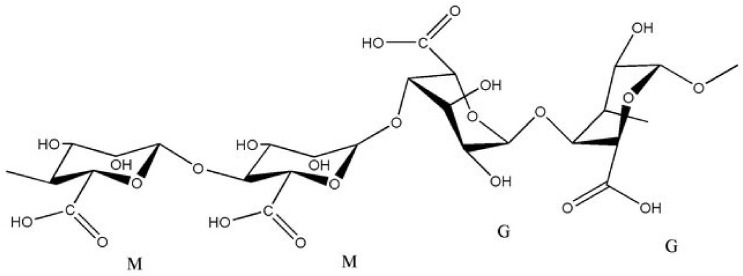
Chemical structure of alginate (M and G blocks). Reproduced with permission from [43]. Copyright 2020 IOSR Journal of Pharmacy.

Alginate is commonly used to manufacture hydrogels due to its ability to retain water. This linear polysaccharide is obtained from brown seaweed or bacteria. The quantity and quality of alginate extracted from brown seaweed may depend on the species, as well as the harvest season. Typically, the amount of extracted polysaccharide is up to 40% of the dry matter of brown seaweed and also strongly depends on the extraction method [17,36,37]. Alginate consists of two hexuronic acids—α-L-guluronic (G) and β-D-mannuronic (M), linked by 1–4 glycosidic bonds, as shown in Figure 2 [20,38]. Ionic gel formation depends on the chemical composition and block distribution in the alginate molecule [17,28]. The physical properties are determined by the numbers of MM, GG, and MG blocks. The high viscosity is dependent on the high content of M blocks, while the high content of G gives higher gelling properties [39]. Alginate-based gels can also be evaluated using the M/G ratio. Flexible gels are obtained using a high M/G ratio, while brittle gels are obtained from sodium alginate with a low M/G ratio [40]. Although alginate is biocompatible, biodegradable, and non-toxic, it has several disadvantages, such as low its bioadhesivity and biological inertness, which limit its applications, particularly in tissue engineering. The main drawback of alginate is the difficulty of obtaining pure alginate from contaminated marine algae. Removal of impurities is possible through further purification steps, which generate additional costs and are time-consuming [41,42].

In order to increase the mechanical properties of alginate hydrogels, a variety of covalent cross-linking methods have been used. However, the mechanical properties of ionically cross-linked alginate hydrogels decrease over time on account of external streams of cross-linking ions into the surrounding environment [44]. The mechanical properties of alginate-based hydrogels may also depend on the gel homogeneity, which can be controlled by the gelling rate. An important parameter that affects the gelling rate is the temperature of gelation [45]. The combination of alginate hydrogels with other materials can lead to the improvement or changes of their physical and mechanical properties [46].

Alginates are available in various forms, including hydrogels, microcapsules, microspheres, fibers, foams, and sponges. However, the most commonly used form, especially in the medical field, is the hydrogel form [46]. The rheological properties of the alginate hydrogel and its ease of gelation, biodegradability, and lack of toxicity make it suitable for a wide range of industrial applications (e.g., in the food industry, agriculture, cosmetics, packaging, and textiles). Its biomedical applications include dentistry, pharmaceuticals, wound healing, drug delivery, cell therapy, and as a bone graft substitute for spine fusion [4,47]. Fabrication of this hydrogel in the medical industry requires the use of high-purity sodium alginate to prevent immunogenicity [28,29].

### 2.2. Gelatin

**Figure 3:**
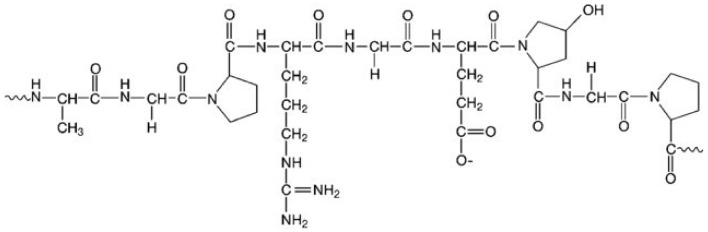
Chemical structure of gelatin. Reproduced with permission from [50]. Copyright 2020 John Wiley & Sons.

Gelatin is a type of protein manufactured through hydrolysis of collagen of animal origin (i.e., bovine, porcine, or fish collagen), connective tissues, and bones [32,48]. Its biocompatibility, lack of immune responses in the body, degradability, and absence of toxicity resulted in its approval by the U.S. FDA. Thus, it is used in the food industry, as well as in the biomedical sector (e.g., pharmacy, wound healing, drug delivery, gene therapy, tissue engineering, regenerative medicine). It is one of the most often utilized materials for cell cultures due to its retention of collagen’s bioactive sequences. This enables the creation of a suitable microenvironment for cell adhesion, migration, proliferation, and differentiation [17,32]. Despite its numerous advantages, gelatin also has some drawbacks. One of them is its solubility, because at around 30–40 °C it passes from a gel state to a solution, which limits its long-term use in transplantation. In order to prolong the degradation time and increase the water resistance of gelatin, it is necessary to apply cross-linking [49]. In Figure 3, the basic chemical composition of gelatin is shown.

Gelatin is exposed to factors such as temperature, pH changes, and the presence of solvents, which modify its flexibility, meaning it takes various forms. Below 40 °C it can occur in a gel state (the reaction is thermally reversible), while above this temperature it can become an aqueous solution. Chemical modifications and cross-linking change the structure of gelatin hydrogel, as well as its properties and the forms it acquires [32,51].

Depending on the animal species, gelatin may have different properties. Wang et al. [52] provided a comparison of methacrylate gelatin from cold water fish and pigs and cold-soluble gelatin. Cold-soluble gelatin has a balance between thermal stability and physical and biological properties compared to the other types of gelatin. Cold water fish gelatin also has thermal stability when compared to pork [52]. A comparison of the properties of gelatin from cold water fish and pigs was also made by Yoon’s [53] research team. The research showed the same results as in Wang’s study [53].

### 2.3. Alginate Dialdehyde-Gelatin Hydrogels

**Figure 4:**
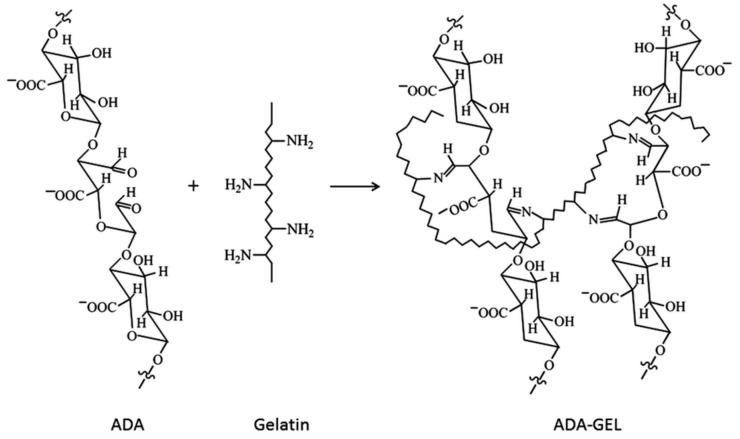
Chemical structure of alginate dialdehyde-gelatin (ADA-GEL). Reproduced with permission from [51]. Copyright 2020 Royal Society of Chemistry.

Lately, there has been a lot of interest in hydrogels, which are a combination of natural polysaccharides (e.g., alginate) and proteins (e.g., gelatin). This is due to the adverse properties of alginate itself, such as its inefficient cell attachment, poor alginate–cell interactions, and slow degradability with uncontrolled kinetics [54]. The solution to these limitations may be the use of alginate that has been previously oxidized to form alginate dialdehyde (ADA) and which is subsequently covalently cross-linked with gelatin (GEL). Between aldehyde groups of ADA and free amino groups of gelatin, Schiff’s base is formed [51,55,56]. The chemical structure of ADA-GEL is shown in Figure 4.

The obtained alginate-gelatin cross-linked hydrogel (ADA-GEL) can be used to produce microcapsules (for encapsulation of bioactive compounds or cells and for drug delivery) [56,57,58] and can be applied as a non-cytotoxic biomaterial with good mechanical strength and biocompatibility in regenerative medicine (e.g., bone tissue regeneration) [55], or as a soft tissue adhesive in wound healing [59].

The obtained alginate-gelatin cross-linked hydrogel can differ in its microstructure and physico-chemical properties, depending on the oxidation degree of the ADA and the cross-linking degree and gelation time of the ADA-GEL [51,55,59]. Microcapsules produced from ADA-GEL are characterized by their higher degradability when compared to alginate microcapsules [51], and exhibit good cell adhesion, proliferation, and migration properties [51,57,60]. An interesting application of alginate dialdehyde cross-linked gelatin hydrogel was suggested by Manju et al. [61]. This hydrogel was used as a coating material for polyethylene terephthalate (PET) vascular graft prostheses, which are intended to replace compromised arteries in the body. The ADA-GEL was non-toxic and was characterized by its biocompatibility, biodegradability, and reduced water permeability, while it had no effect on the mechanical properties of the graft and improved the adhesion and proliferation of fibroblast cells.

ADA-GEL can be also used as an ink, but this application may be limited due to its viscosity [58]. Nevertheless, there are some studies using ADA-GEL as an ink in 3D printing technology. In the work by Dranseikiene et al. [58], ring-shaped structures containing alginate dialdehyde-gelatin cell-laden hydrogel were fabricated. The cell viability (MG-63 osteosarcoma cells) in samples depended on the cross-linking agent (CaCl_2_ or BaCl_2_), with better results in terms of increased cell viability and cell network formation being observed after cross-linking with Ba^2+^. Ruther et al. [60] investigated the printing of three-dimensional, cell-containing, vessel-like structures using ADA-GEL ink. This artificial vessel supported the viability, proliferation, and migration of immobilized cells (human dermal fibroblast and human umbilical vein endothelial cells).

## 3. Material Additives Used in Gel Systems

Apart from the basic materials forming the gel systems, additives are also used to increase their efficiency. Material additives are inactive ingredients designed to structurally improve and enhance the hydrogel’s characteristics, as well as influence the degradation profile and interactions between cells and the matrix. Among the additives, materials from natural (e.g., collagen, pectin), synthetic (e.g., polyvinyl alcohol, poloxamers), and semi-synthetic sources (e.g., cellulose derivatives) can be distinguished. The used materials include peptides that increase cell adhesion, nanomaterials that adjust rheological properties to specific applications, as well as growth factors that enable direct stem cell differentiation [62]. Additives added to alginate hydrogel culture media are used to influence cell growth and attachment. Furthermore, they improve resistance to degradation and facilitate the production of the hydrogel, which is a benefit during additive manufacturing and processing in terms of bioprinting [63].

To enhance the strength and increase the mechanical properties of hydrogels when using additive manufacturing technologies in tissue engineering, additives in the form of microfibers, nanofibers, microparticles, and nanoparticles, as well as woven and non-woven scaffolds, are also used. Reinforcement of hydrogels in this manner eliminates the need for dense cross-linking, allowing them to obtain similar mechanical properties, thereby improving cell migration and promoting the formation of neo-tissues [64]. The nanomaterials included in hydrogels, such as cellulose and chitin, can affect the structures of the hydrogels, thus improving their mechanical properties and drug release profiles [65]. Zhang et al. [66] created hydrogel composites with alginate microcapsules cross-linked with Ca^2+^ ions and cellulose whiskers. As a result, the mechanical properties of the hydrogels were improved and the release profile of the drug was more stable, without causing cytotoxicity. In Kinneberg’s work [67], the application of a microporous, fibrous collagen scaffold increased the tangent modulus and toughness of the hydrogel. Research carried out by Park et. Al. [68] showed that the addition of nanocellulose to alginate hydrogel enhanced its mechanical strength, but also improved the cells’ ability to proliferate and prolonged their viability. Similar research was carried out by Siquiera et al. [69], whereby the viability of L929 fibroblast cells was examined in alginate hydrogels and compared to nanocellulose–alginate hydrogels containing nanofibers and nanocrystals. The addition of nanocellulose supported the cell bioadhesion and cell growth on the created medium. Nanocellulose is one of the materials that improves not only the mechanical properties of the created substrates, but also has a direct effect on cells and their development.

### 3.1. Hydrogel Fabrication from Materials Useful for 3D Cell Culture

Various materials can be used to create different types of hydrogel matrices. The compositions of these media depend on the type of culture and the results expected by the researchers. There is no doubt that 3D cultures and systems are more reliable setups that faithfully recreate cellular responses. The available data show that 3D cells do not have receptors that recognize alginates. Having high purity is of special importance, because some residual endotoxins can interact with receptor CD14 (a receptor for complexes of lipopolysaccharide and lipopolysaccharide binding protein) [28]. Thus, highly purified alginates are crucial in future applications. Natural substrates such as sodium alginate have been used by Zimmermann et al. [70], Dvir-Ginzberg et al. [71], and Bauer et al. [72]. It was also noted that alginates can be functionalized to obtain specific properties. For instance, it was found that arginine–glycine–aspartic acid conjugated to sodium alginate (RGD-alginate) can stimulate cell proliferation and can affect cell adhesion and differentiation [73]. De Melo et al. [74] investigated PEG poly(ethylene glycol) and sodium alginate concentrations to optimize a polymer blend for usage as a mechanically resistant support hydrogel. It was also shown that a combination of alginate with gelatin can decrease gelatin leakage over prolonged culture while simultaneously enhancing cell adhesion and the secretion of vascular endothelial growth factor (VEGF) [57]. Chung et al. [75] and Li et al. [76] prepared a sodium alginate-gelatin hydrogel in order to examine cell development. The gelatin strengthened the cell-filled sodium alginate scaffolds. Demirtaş et al. [77] fabricated sodium alginate and alginate-hydroxyapatite (HA) hydrogels and used them for printing. Schwarz et al. [78] manufactured 3D-printed, grid-like structures for cartilage tissue engineering by using a system composed of an oxidized alginate-gelatin hydrogel. The examples of the applied materials for the 3D cell culture substrates and their concentrations are shown in Table 1.

According to the type of cell, different conditions are needed for growth, proliferation, or differentiation, and therefore a surface is required for culture, which will provide the cells with a specific environment. Alginate-gelatin-based hydrogels are mainly found as substrates in laboratory studies, however there are already commercially available cell culture substrates made from other materials, which are intended for specific cell types. One of them is Matrigel, a gelatinous protein mixture obtained from Englebreth–Holm–Swarm tumor cells from mice. It is used as a basement membrane matrix for stem cells and maintains them in an undifferentiated state. However, this substrate is not precisely defined, thus it may not reflect the accuracy of the research results [87]. Similar to Matrigel is Cultrex^®^ BME, which is used as its substitute. Cultrex^®^ BME is also a homogenate of mouse tumor tissue, therefore both substrates differ in composition from human transmembrane proteins [88]. HyStem^®^-C is a biodegradable and biocompatible material consisting of three blocks: Glycosil^®^ (thiolated hyaluronic acid), Gelin^®^ (tioltated gelatin), and Extralink^®^ (polyethylene glycol diacrylate). It is capable of mimicking ECM, and therefore is used as a tissue model in developing new drugs and cell cultures. It is a suitable material for bioprinting and for designing organ models [89]. Another hydrogel suitable for cell culture is Biogelx™, obtained from synthetic peptide powder functionalized with collagen, fibronectin, and laminin, which was created to mimic the ECM. PuraMatrix™ is a peptide hydrogel consisting of amino acids that is suitable for 3D cell culture [6]. Most of these products are used for research purposes. However, new materials for 3D cell cultivation and bioprinting, such as bacterial cellulose–gelatin hydrogels [90]; synthetic, thermo-responsive polyisocyanopeptide hydrogel [91]; chitosan–fibrin hydrogel for tissue engineering [92]; collagen-based hydrogels [93]; and others are currently being fabricated and tested.

### 3.2. Hydrogel Cross-Linking Methods

Cross-linking is a process that leads to the formation of a three-dimensional polymer network structure by binding one polymer to another. It is a stabilization process—the attached polymers lose their ability to move as a single chain. The consequence of cross-linking is a change in the physical and chemical properties of a substance. Cross-linked polymers are mechanically stronger but less flexible and less heat- or solvent-resistant in comparison to liquid polymers [94].

Cross-linking of hydrosols (liquid polymers) has positive effects on the physico-chemical properties of hydrogels—including their water holding capacity, hardness, and structural integrity, which affect their cytocompatibility—as well as on their biochemical properties [94,95]. In bioprinting, the cells are mixed in hydrosol and the cross-linking of the hydrogel takes place after extrusion from the bioprinter. Thus, the cross-linker should not be toxic to cells and should have fast cross-linking capability to maintain the highest cell viability [96].

**Figure 5:**
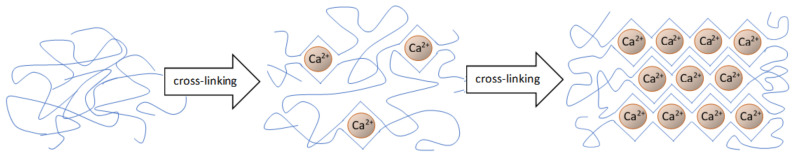
Graphical illustration of ionic cross-linking of polymer chains (own source).

Sodium alginate is most often physically cross-linked through the application of ionic interactions [45,94,95]. Cross-linking with divalent or trivalent ions occurs under mild conditions, at room temperature and neutral pH [94,96]. Such cross-linking conditions are suitable when using cells. Cross-linking of sodium alginate occurs through a bond between G blocks (guluronate blocks) and divalent ions. This is an “egg box” cross-linking model [45,95]. Ca^2+^, Ba^2+^, and Zn^2+^ are divalent ions, which are usually used in such applications [45,95,96]. An example of ionic cross-linking is presented in Figure 5. The choice and concentration of ionic cross-linkers is a critical factor in order to obtain enhanced cell viability, better printability, and mechanical stability of a hydrogel during bioprinting [96]. Other studies have shown that the subsequent addition of cross-linkers in appropriate ratios can improve the cell viability and mechanical stability of hydrogels [96,97,98]. The stability of hydrogel cross-linking in cell cultures is an important issue. Under physiological conditions, divalent ions can be exchanged with monovalent cations, which contribute to the depletion of the network [45].

Photo cross-linking is another covalent cross-linking approach. Modified sodium alginate with the addition of methacrylate can be cross-linked via exposure to an argon ion laser (514 nm) for 30 s in the presence of eosin and triethanolamine. Cross-linking takes place in mild conditions with the proper selection of chemical initiators. This method can be used directly in contact with drugs and cells. To avoid harmful cross-linking conditions (where a light sensitizer or acid release is required as a by-product), polyallylamine partially modified with acetylphenoxyinninamyldiene chloride is used as an alternative, which converts to a dimer under the influence of light at about 330 nm and does not release toxic by-products during the cross-linking reaction [45,99]. Additionally, using light radiation, the resulting hydrogel from sodium alginate and polyallylamine has improved mechanical properties [45].

Thermal gelation is the next cross-linking approach. The use of this method for sodium alginate is not common due to the poor sensitivity of sodium alginate to temperature. However, semi-interpenetrating polymer networks (semi-IPN) can be used. An example is a semi-IPN prepared from copolymerization of N-isopropylacrylamide (NIPAAm) with a poly(ethylene glycol)-co-poly(-caprolactone) (PEG-co-PCL) macromer in the presence of sodium alginate using UV irradiation. The use of sodium alginate in semi-IPNs improves the mechanical strength. The increase in hydrogel swelling with the increase in the concentration of sodium alginate at a constant temperature and decrease in swelling with the increase in temperature can be used for drug delivery [45,100].

**Figure 6:**
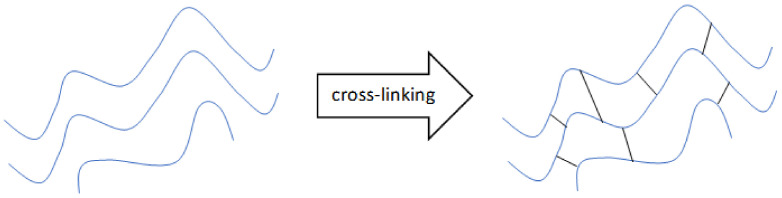
Graphical illustration of covalent cross-linking of polymer chains (own source).

Covalent cross-linking is another method. Covalent networks are formed by polymerization of end-functionalized monomers or macromers (Figure 6) [94]. In this type of network, cross-linking dissociation and bond transformation do not occur. Water migration causing stress relaxation is also preserved. Chemically modified sodium alginate is used for covalent cross-linking [28]. One of the covalent cross-linkers used in the cross-linking of sodium alginate is poly(ethylene glycol) (PEG), which is used for PEG-diamines. It has been shown that the mechanical properties and swelling can be controlled by using covalent cross-linking agents. Using hydrophilic cross-linkers can compensate for the lack of the hydrophilic nature of the gel due to the cross-linking reaction [45].

An interesting approach to create cross-linked hydrogels is to use the cells themselves. For example, cells added to adhesive ligand-modified sodium alginate (arginine-glycine-aspartic acid peptide sequence-modified (RGD-modified)) can form networks by binding multiple polymer chains based on the receptor–ligand binding. This method of cross-linking occurs without the addition of cross-linkers [45,101].

Gelatin, as a product of collagen hydrolysis, can be cross-linked with transglutaminase, which catalyzes the acyl transfer reaction between the γ-carboxamide group of peptides or protein-bound glutamine residues (acyl donors) and primary amines (acyl acceptor) containing ε-amino groups of lysine. A network of isopeptide bonds is formed [102]. This approach was used in research conducted by Schwarz et al. [78] and Chen et al. [95]. The properties of interpenetrating alginate-gelatin hydrogel have been studied. Sodium alginate has been cross-linked with calcium ions, while gelatin has been cross-linked with transglutaminase [95]. Another gelatin cross-linking agent could be water-soluble 1-ethyl-3-(3-dimethylaminopropyl) carbodiimide (EDC), which specifically forms amide or peptide bonds between the carboxylic acid and protein amino groups [103].

One way to cross-link gelatin is to modify it with tyramine (TA). TA–gelatin is obtained by carbodiimide coupling of tyramine groups on gelatin. Then, TA–gelatin is covalently cross-linked in the presence of horseradish peroxidase (HRP) and hydrogen peroxide (H_2_O_2_). Tyrosine–tyramine, tyrosine–tyrosine, and tyramine–tyramine bonds are formed. The addition of TA–gelatin increases the mechanical stability and gelation rate and slows the enzymatic degradation of silk hydrogels cross-linked in physiological buffers. Manipulation of the amount of TA–gelatin added allows modulation of the silk hydrogel’s bioactivity, in turn affects the cell’s microenviroment [104].

### 3.3. 3D Cell Cultures

Cell cultures growing in the three-dimensional form behave quite differently than in a monolayer. Due to the use of 3D culture, the impact of cell interaction and the influence of the external environment on cell development have been investigated. This is why the research on three-dimensional cell cultures is so important. In vitro experiments aim to reflect the in vivo conditions to the greatest extent. Each cell line has individual requirements and conditions that must be met to achieve the most reliable test results. It is not realistic to work out a uniform template of optimized culture conditions for all cell lines. However, for faster optimization of three-dimensional cell culture conditions, it is worth paying attention to certain aspects described below. This review focuses on the use of 3D-printed alginate-gelatin hydrogel scaffolds in three-dimensional cell cultures.

Hydrogels obtained from sodium alginate and gelatin imitate the extracellular matrix. Both components of the gel, which are cross-linked under mild conditions, are transparent; decomposition of the network can take place under controlled conditions; and the porosity of the material provides the cells with access to nutrients and metabolite exchange [28,105]. It is worth noting that gelatin as an animal-derived material could be an immunogenic agent. However, the use of sodium alginate alone does not provide optimal conditions for the development of culture [28,106].

Depending on the application of the hydrogel, the selection of the type and concentration of sodium alginate and gelatin affects the properties of the hydrogel and the cell growth [28,107]. The number and length of G blocks that are involved in ion cross-linking depend on the porosity, stability, and elasticity of the gel [28,108]. The mechanical properties can affect cell proliferation and conditions.

Alginate has different affinities for divalent ions, which is why their selection can affect such properties as swelling, stability, and cell growth [28]. Sarker et al. [96] studied the effects of three ionic cross-linkers of alginate—CaCl_2_, ZnCl_2_, and BaCl_2_—in terms of the viability of Schwann cells (the main glial cells of the peripheral nervous system) and the mechanical properties of hydrogels. Research showed that the best of these three cross-linking ions in terms of the impact on mechanical properties was Ba^2+^, while Ca^2+^ had the best effect on cell viability. The research team suggested using both cross-linking compounds, namely CaCl_2_ and BaCl_2_ [96].

When forming hydrogels for cell cultures, an important aspect is sodium alginate modification in order to adapt and control the type of and interaction between cells. Examples of such modifications are the covalent attachment of heparin-binding peptides (HBP) [28,109] or peptides found in extracellular matrix proteins [28,109,110]. In a ligand–receptor approach, cells can interact with the hydrogel network. For example, attachment of the RGD (arginine–glycine–aspartic acid) peptide sequence has also been shown to affect the initiation of interactions with cells via recognition of an adhesive molecule (RGD sequence) by cell receptors. Some cell lines may show accelerated proliferation when sodium alginate is modified by the RGD sequence [28,111]. As it turns out, the sequence fragments flanking the RGD and the conformation (linear or cyclic) and length of the RGD sequence all impact the integrin receptor affinity and selectivity, depending on the cell type. Additionally, depending on the cell type, the used RGD sequence density may affect the development of the culture [28,112].

An important issue when establishing a 3D cell culture is the printing process. Attention should be paid to parameters such as the rheological properties, printing process, and needle diameter relative to time; the dimensions of the printing thread; and the impact on cell survival. Currently, computational simulation can be used to implement process design strategies, as well as comparisons with other inks [113].

Table 2 shows a comparison of studies on alginate and gelatin-based hydrogels in three-dimensional cell cultures. Depending on the composition of the hydrogel, the modification strategy, and the cross-linking methods used, different concentrations of alginate and gelatin were used. Based on these few examples, it can be seen that studies on three-dimensional cell cultures have been carried out on different cell lines.

**Figure 7:**
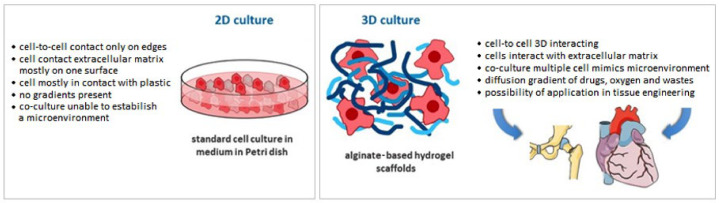
Comparison of 2D culture and 3D model based on alginate hydrogel (own source).

Alginate-hydrogel-based cell constructs are good candidates for tissue engineering and regenerative medicine applications (Figure 7). The available studies indicate that stem cells can be combined with biomaterial scaffolds for in vivo and in vitro research. There are various biomaterial formulations for tissue engineering, e.g., hydrogels, electrospun nanofibers, and microspheres. Alginate material is recommended for hydrogels and electrospun nanofibers [119]. This material was efficiently used with adipose-derived adult stem cells (ASCs) and bone marrow-derived MSCs (mesenchymal stem cells) to generate replacement cartilage [120] and was also used with neural tissue [121]. In another study, the authors blended calcium alginate gels for internal and external gelation. The gels were injectable and could be applied as immobilization matrices for human MSCs [122]. Pandolfi et al. [123] verified that alginate-based microencapsulations can be efficiently applied for transplants in patients that are not eligible for immunosuppressive therapies. Consequently, alginate-based materials seem to be good candidates for tissue engineering and regeneration.

Clinical applications for the materials will be possible in the relatively near future. The application of 3D hydrogels in clinical trials is still in progress, however there are some available data that show that they could be alternatives in regeneration or transplantation treatments. Forte et al. [124] developed a composite hydrogel based on gelatin to form brain tissue phantom . The authors suggested that this newly developed model will be useful in traumatic brain injury (TBI) studies. Gelatin-based hydrogels are used as extracellular matrices (ECMs) in 3D bioprinting technologies [125].

Alginate has been used in wound-healing processes in the form of electrospun hydrogels and sponges [126,127]. The authors of a previous study observed a significant improvement of the healing process and monocyte stimulation, as well as higher cytokine levels, such as tumor necrosis factor-α and interleukin-6 [128]. It was also proven that alginate might be useful in reconstructive surgery and bone regeneration, e.g., alginate scaffolds helped in bone tissue formation [129].

Alginate was also used in cardiac muscle regeneration in the form of a hydrogel and porous 3D scaffold [130]. The most advanced clinical trials involving alginate-based systems for cardiac regeneration have included Algisyl-LVR™ sponsored by LoneStar Heart, Inc (Laguna Hills, CA, USA), and PRESERVATION 1 sponsored by Bellerophon BCM LLC (Hampton, NJ, USA). It is also noted that alginate implants are under clinical investigation for intramyocardial delivery in patients with acute myocardial infarct (MI).

There are reports of clinical trials concerning the utility of gelatin with selected renal cells in patients with type 2 diabetes [131] and chronic kidney disease, and of gelatin with renal autologous cells in patients with chronic kidney disease from congenital anomalies of the kidneys and urinary tract [132].

## 4. Mechanical Properties of Alginate-Based Hydrogels

The mechanical properties of sodium alginate hydrogels depend on the number and sequence of monomer units (M and G), as well as their molecular weight, chain stiffness, and cross-linking density [133,134]. Similarly, the mechanical stiffness of ion-cross-linked sodium alginate hydrogels depends on the molecular weight of the sodium alginate and the concentration of binding cations [135]. There is a relationship between the rheological properties of the pre-gel solution and the mechanical stiffness of the post-hydrogel with the particle concentration and molecular weight distribution of the polymer used to form them [133].

The mechanical properties of biomaterials are an important parameter in regulating the behavior of cell adhesion. Indeed, cells can remodel the mechanical, structural, and chemical composition of the surrounding ECM. It is also known that the mechanical properties of the extracellular microenvironment change the behavior of cells, including their spreading, proliferation, or differentiation [136]. Most studies have focused on the influence of matrix stiffness on cells using hydrogel substrates, which show purely elastic behavior. Examples of mechanical properties for the most commonly used hydrogels are given in Table 3. Importantly, the matrix stiffness influences the ability of stem cells to differentiate towards specific lineages. Mesenchymal stem cells differentiate towards a neurogenic lineage when cultured on soft 0.1–1 kPa substrates, towards a myogenic lineage when cultured on 8–17 kPa substrates, and towards an osteogenic lineage when cultured on stiffer 25–40 kPa substrates [137]. Besides the stiffness of the matrix, the viscoelasticity and stress relaxation are also important [72].

Commercially available sodium alginate reaches molecular weights in the range of 32,000–400,000 g/mol. A decrease in the pH of the sodium alginate solution results in increased viscosity (reaching a maximum around pH 3–3.5). The shear modulus of the hydrogel depends on the sodium alginate concentration [133]. The physical properties of the obtained gel can be improved by increasing the molecular weight of the sodium alginate. Solutions made from high molecular weight polymers of sodium alginate become more viscous, which is often unsatisfactory during processing [138]. The mechanical strength also depends on the sources from which the alginates are obtained, e.g., *Azotobacter* alginates have comparatively high stiffness [139]. The viscosity of the pre-gel solution and its stiffness after gelation can be successfully controlled through manipulation of the molecular weight and distribution [133].

## 5. Alginate-Gelatin Hydrogel as an Ink in 3D Bioprinting

Additive manufacturing is a technology that could be widely applied in biomedicine. Materials and technologies used in medical and biological fields have to fulfill specific requirements. Bioprinting technology is a branch of additive technology, commonly known as 3D printing, which is utilized as an innovative tool in manufacturing living functional tissues from cells and supporting biomaterials as bioinks in computed printing systems. This technology enables a wide range of applications in tissue engineering or regenerative medicine, for example replacing injured or damaged tissue manufactured in an automated way with high repeatability [152]. Moreover, 3D printing, including bioprinting technology, consists of sequential printing with precursor bioink (which consists of a polymer or composite) in a predefined trajectory and within cells. Multilayer ink deposition enables controlled spatial positioning of cells, thus facilitating the precise creation of scalable structures, which cannot be provided by two- and standard three-dimensional cell cultures [153]. Bioink is a specially prepared ink for biological and medical applications that is non-toxic, enabling cell and tissue viability. Bioink is composed mainly of hydrogel due to its biodegradability and ability to mimic the anatomy and mechanical features of the body, however additionally it contains living cells, cell aggregates, or bioactive molecules, creating entirely biologically functional products. The potential application and cell type determine the choice of bioink and bioprinter to be adopted [28,152].

**Figure 8:**
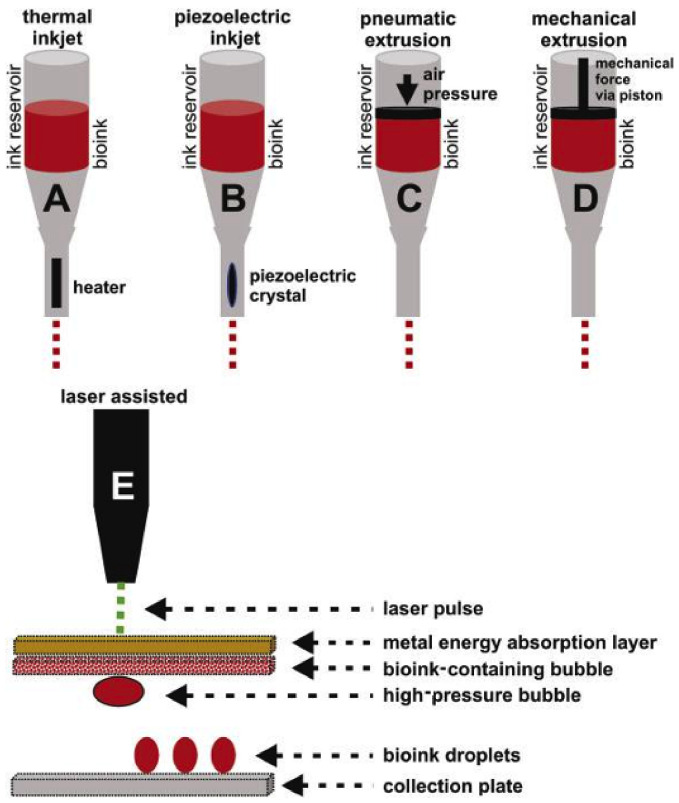
Schematic representation of the most common methods of bioprinting. (A) Thermal inkjet bioprinting. (B) Piezoelectric inkjet bioprinting. (C) Pneumatic extrusion bioprinting. (D) Mechanical extrusion bioprinting. (E) Laser-assisted bioprinting. Copyright 2017, Chongqing Medical University. Production and hosting by Elsevier B.V [154]

The widely used 3D printing methods for plastics, metals, and ceramics are not adequate or appropriate for biomaterials applied in cell culture media. Conventional 3D printing strategies described by Bishop [154], such as inkjet printing, microextrusion, and laser printing, have also been developed to generate 3D biological structures directly surrounding cells and bioactive agents, including various biomimetic tissues, e.g., bone tissue, cartilage and osteochondral tissue, heart tissue, blood vessels, liver tissue, and tumors [155,156]. Over the last decade, there has been tremendous progress in the development of three-dimensional bioprinting. The most commonly used 3D bioprinting techniques include inkjet printing or droplet bioprinting, microextrusion, and laser-assisted bioprinting (LaBP) [152,157] (Figure 8).

Three-dimensional bioprinting is a versatile technique for creating biomimetic tissue structures [23]. The production of alginate substrates using the additive method is an excellent choice due to the uncomplicated processing procedure and reduction of waste, which affect the material yield and programmable viscosity (ranges from 0.03 Pa/s to 6 × 10^4^ Pa/s). However, in addition to its accuracy, the procedure is limited by its resolution (200 µm) [158]. The printability of alginate bioinks is dependent on their viscosity, however 3D biostructures exhibit constant rigidity in relation to certain conditions [153].

Bioinks have to be suitable for a range of key requirements for processing with a 3D bioprinting machine. Printability should be determined by rheological properties, bioink consistency measurements, and sample dimensions [159,160,161]. The most important feature of the rheological properties is the bioink viscosity, which can directly affect the printing pressure required for the material dosage, and thus the cell viability in bioink and shape fidelity of the printouts, as well as the elastic recovery, viscoelastic shear moduli, and shear stress. Excessive pressure in the printing nozzle can damage the cells during printing, thus it is important to control shear stress during extrusion because the cells suspended in the ink have differing sensitivity [162,163]. Gao et al. [162] examined the rheological properties of an alginate-gelatin hydrogel and their influence on the bioink and cells placed in it during the manufacturing process. In a study carried out by Diamantides et al. [164], the effect of the density of cells contained in a hydrogel on its rheological properties was examined. Another factor that may affect the rheological properties of some hydrogels is temperature. The extrusion of bioinks during using 3D bioplotters does not require increased temperature, and therefore does not affect cell destruction either. Such bioinks, which are exist sol form or are gelated after being printed from the nozzle, are not suitable for bioprinting because the mechanical strength of the structure is not sufficient to support the cell culture. However, when bioink is too viscous, the filament is irregular and unstable during printing. The manufactured structure requires the use of a semi-viscous hydrogel to maintain the integrity of the structure consisting of regular fibers, while for the highest cell viability, the viscosity of the bioink should be as low as possible to reduce the induced shear stress [159]. It is possible to increase the viscosity by applying higher pressure on the hydrogel extrusion, which eliminates the gel outflow from the nozzle and provides higher accuracy to the printed structures [161].

The efficiency of bioprinting depends on both the bioink printability and the achieved cell viability, as well as the process parameters (e.g., printing speed, resolution, pressure) [165,166]. The accuracy of the obtained structure is also affected by the solidification of the bioinks during the bioprinting process, which usually takes place through cross-linking. The gentle solidification of the printout is the main prerequisite for the selected bioinks, as they cannot cause damage or cell death [166]. Certain 3D printing applications (e.g., when building more complex structures for tissue regeneration) do not provide the smart response needed for such an artificially created structure to properly work in the body, creating the opportunity to utilize 4D printing [167].

Sodium alginate hydrogel is widely used in the bioprinting process due to its biocompatibility, rapid ability for cross-linking, and simple regulation of its mechanical properties through physico-chemical modifications [156,168]. Due to its hydrophilic nature, alginate scaffolds allow the cell viability to be maintained during the culture, because media and cells are pulled into the scaffold via capillary action. One of the benefits of possessing dense cell cultures is the maximization of the contact interactions between cells [165].

Sodium alginate is not suitable for direct printing because the structure obtained is not rigid enough and takes a liquid form after printing. Recently, multi-material bioinks have been used more frequently, as these combinations can benefit from the advantages of different materials [160]. However, it is possible to strengthen the structure of sodium alginate by adding gelatin while maintaining its biocompatibility, because dissolved gelatin forms a solution at body temperature (37 °C) and can form a gel after cooling (<29 °C). These thermal properties of gelatin guarantee good flow characteristics for bioinks and allow quicker gelation during the bioprinting process compared to alginate combined with Ca^2+^, which improves the initial stability of the manufactured structure [75,153,169]. One well-known and accessible composite bioink is based on a combination of alginate and gelatin. Alginate allows fast cross-linking, whereas gelatin provides thermoresponsive properties [160]. Alginate-gelatin bioink combines the rapid ionic cross-linking of alginate and the temperature sensitivity of gelatin for multilayer printing and is widely used as bioink in cell bioprinting applications [159]. The substrate properties can also be modified by using different solvents and gelatin concentrations. Their suitability for printing can also be supported by adding an extruder heating system. The rheological properties of alginate-gelatin hydrogels are influenced by temperature, in addition to the concentrations of both substances. Temperature should not lead to cell apoptosis in the culture [161]. Other materials used to support the biological activity of alginate-based bioinks include fibrin, which improves interactions with cells, as well as polyvinyl alcohol and hydroxyapatite (HAp), which enable multicomponent bioinks to be obtained. Cellulose can also be used, which provides a bioink with appropriate shear-thinning action and can accurate reproduce the shapes of the created structures [156].

Alginate, which has a higher molecular weight and is highly concentrated, is a shear-thinning physical gel. It is important to adjust its concentration in order to obtain the appropriate properties when printing. There are ways to process unprintable alginate compositions using different cross-linking strategies, such as printing on calcium chloride solutions, spraying calcium chloride mist onto the solution coming out of the nozzle, or pre-cross-linking with calcium chloride [160].

Traditional 3D structures manufactured using 3D bioprinting technology with static mechanical properties might not be suitable for biomedical applications [170]. A new solution, which can more accurately mimic the dynamics of native tissues, has recently been developed using four-dimensional bioprinting (4D) technology, based on three-dimensional bioprinting with additional shape-shifting capability [171]. It is possible to design biologically active structures that respond to impulses, and after appropriate stimulation, may change their shape to achieve the required functionality. Programmable bioinks capable of being used in 4D bioprinting are being produced to obtain various stimulus-responsive biomaterials, including thermal, humidity, electrical, magnetic, and photo-sensitive materials [172].

## 6. Conclusions

Sodium alginate in the form of hydrogel is a low-cost biomaterial that demonstrates good printability and excellent biocompatibility. This is why it is used in a wide range of applications, such as in wound healing, regenerative medicine, and drug delivery, as well as for cell culture. Alginate-gelatin hydrogel has very beneficial properties; the combination of these two substances provides a substrate with mechanical properties and a structure adapted to free cell growth. While sodium alginate is a less rigid structure, gelatin increases the rigidity and provides a substrate with a precise shape. Bioprinting technology offers promising opportunities for cell cultures. It allows the preparation of media with specific structures and mechanical properties using a variety of substances.

The production process providing accurate structures using alginate-gelatin bioink in additive manufacturing technology enables the creation of hydrogel substrates of any shape and high porosity, imitating the ECM. This facilitates the correct ingrowth, proliferation, and differentiation of cells outside the body, which will significantly contribute to the development of tissue engineering and regenerative medicine. In vitro 3D cell cultures will enable the future preparation of tissues or parts of organs and could even be an opportunity for drug testing systems.

## Figures and Tables

**Table 1:** The examples of hydrogel materials based on alginate and gelatin used for the production of 3D cell culture substrates.

Hydrogel	Type of Cells	Sodium Alginate Concentrations (w/v%)	Gelatin/Other Additive Concentrations (w/v%)	The Aim of Research	Reference
sodium alginate	C2C12 mouse myoblasts	2.5		testing the influence of substrate stress–relaxation on the regulation of muscle cell (myoblast) proliferation and spreading in vitro	[72]
sodium alginate-gelatin	BL6 primary mouse myoblasts	1, 2, 4	10	optimization of the bioink consistency and investigation of printability with live cells	[75]
sodium alginate-gelatin	rat Schwann cell line RSC96s	4	20	examination of the cell behavior in the created microenvironment	[76]
sodium alginate-gelatin	L929, mouse fibroblast cell line	1	4	evaluation of the cell viability and possibility to develop in the created microenvironment	[44]
		2–2.5	4–8	analysis of the printability with the cell-filled bioink and optimization of the parameters to obtain structures for cell cultures	[79]
		2.5	8		
sodium alginate-gelatin	Non-Small Cell Lung Cancer (NSCLC) PDX (EGFR T790M) cell line	1–6	3–8	research on the cells growth on the manufactured hydrogel; modification of the alginate-gelatin concentration to achieve good printability	[80]
sodium alginate-gelatin	mesenchymal stem cells (MSCs) from adult sheep adipose tissue	1, 3, 5, 7, 9	6	examination of the printing possibilities according to the individual component concentrations	[81]
		5	2, 4, 6, 8, 10		
sodium alginate-gelatin	human adipose-derived mesenchymal stem cells (MSCs)	3	10	evaluation of the cell development on produced substrates	[82]
sodium alginate-gelatin with mouse plantar dermis (PD)	mouse mesenchymal stem cell (sMSCs)	1	3	investigation of the chemical and physical properties of the Alg-Gel-PD bioink, and its effect on embedded mouse mesenchymal stem cells (MSCs)	[83]
sodium alginate-gelatin mixed with hECM	HepaRG human bipotent hepatic progenitor cells; A549 human epithelial lung carcinoma cells	2	3	the optimization of a bioink composed of hydrogel and human extracellular matrix (hECM) to print human HepaRG liver cells and testing of its suitability for the study of transduction using an adeno-associated virus (AAV) vector and infection with human adenovirus 5 (hAdV5)	[84]
			hECM		
			0; 0.25; 0.5; 1; 2		
alginate dialdehyde-gelatin (ADA-GEL)	osteosarcoma cells MG-63	5	5	evaluation of the cell encapsulation possibility in prepared hydrogel and monitoring of cell activity	[58]
alginate dialdehyde–gelatin (ADA-GEL)	HUVECs human umbilical vein endothelial cells; rat Schwann cells	2; 3; 6	2	characterization of the printability and cell viability of various concentrations of alginate dialdehyde (ADA)–gelatin (Gel) hydrogels for bioprinting	[85]
		2	3; 6		
sodium alginate with d-gluconic acid	rat liver cell	1	0.2	analysis of the cell growth on the created substrate	[71]
sodium alginate/PEO-bisamine (polyoxyethylene bis(amine))	L929, mouse fibroblast cell line	2	5 and 20	evaluation of the cell viability and possibility of developing in created microenvironment	[44]
sodium alginate/PEO with gelatin	mesenchymal stem cells (MSCs)	2, 4, 5	PEO 1:1 with alginate; gelatin: 10%	examination of the cell viability and possibility of proliferating in created microenvironment	[86]

**Table 2:** Comparison of the hydrogel compositions depending on the cross-linking methods and modifications used.

Cell Line	Concentration (w/v%)		Cross-Linking Method		Modification	Reference
	Sodium Alginate	Gelatin	Sodium Alginate	Gelatin		
NB SH-SY5Y *	0.50–0.75	2.00–5.00	Ionic (CaCl_2_)	Covalent (transglutaminase)	n.a.	[95]
1) Hbmsc *; 2) D1 stem cells *; 3) MC3T3-E1 *	2.00	n.a.	n.a.	n.a.	1) linear RGD 2) cyclic RGD	[114]
RSC96 *	1.00–2.50	n.a.	Ionic (CaCl_2_)	n.a.	1) added poly-L-lysine 2) added fibronectin 3) added RGD 4) covalent binding RGD	[115]
ACPCs *	n.a.	8.00	n.a.	1) Free radical polymerization 2) Free radical polymerization and covalent	1) methacrylic anhydride (GelMA) 2) methacrylic anhydride and tyramine (GelMA-Tyr)	[116]
1) HepaRG * 2) A549 *	2.00	3.00	1) Ionic (CaSO_4_)—before printing 2) Ionic (CaCl_2_)—after printing 3) Ionic (CaCl_2_)—during incubation	n.a.	human extracellular matrix (hECM)	[84]
1) Saos-2 * 2) hBMSCs *	4.00	5.00	Ionic (CaCl_2_)	n.a.	1) cellulose nanofiber (CNF) 2) bioactive glass (BaG)	[117]
1) HUVECs * 2) MSCs *	1.00; 2.00; 3.00	GelMA:5.00; 7.00	Ionic (CaCl_2_)	Free-radical polymerization	1) 4-arm poly(-ethylene glycol)-tetra-acrylate (PEGTA) 2) methacrylic anhydride	[118]

Note: NB SH-SY5Y—human neuroblastoma cell line; hBMSC—human bone marrow stem cells; D1—mesenchymal stem cells; MC3T3-E1—osteoblastic cell line; RSC96—neuronal Schwann cell; ACPCs—articular cartilage progenitor cells.

**Table 3:** Mechanical properties of hydrogels.

Material	Example	Tensile Strength	Tensile Modulus	Compressive Strength	Compressive Modulus	Reference
Traditional hydrogel	(PVA), PEG	1 ~ 100 kPa	<100 kPa	10 ~ 100 kPa	1 ~ 100 kPa	[140,141]
Alginate hydrogel	n.a.	~20 kPa	~78 kPa	n.a.	n.a.	[142]
Alginate-gelatin hydrogel	n.a.	~0.5 MPa	~1 MPa	2 ~ 12 MPa	30 ~ 50 kPa	[81,142]
Alginate-gelatin-nanocellulose hydrogel	n.a.	n.a.	~220 kPa	~320 kPa	60 ~ 110 kPa	[143,144]
Nanocomposite hydrogel	PEG/clay	255 kPa	16 kPa	3.7 MPa	38 kPa	[145,146,147]
Cartilage	n.a.	~3 MPa	~9 MPa	~35 MPa	~15 MPa	[148,149]
Collagen fiber	n.a.	~75 MPa	~1000 MPa	n.a.	n.a.	[140,150]
Gelatin hydrogel	n.a.	n.a.	3–25 kPa	n.a.	n.a.	[151]

## Data Availability

No new data were created or analyzed in this study.
